# The contribution of genetics to dental caries, oral habits and occlusal traits in Turkish twins: A comparative study

**DOI:** 10.1186/s12903-023-03426-9

**Published:** 2023-10-09

**Authors:** Esra Oz, Zuhal Kırzıoglu

**Affiliations:** 1 Department of Pediatric Dentistry, Faculty of Dentistry, Suleyman Demirel University, Isparta, Turkey; 2 Private Pediatric Dentist, Isparta, Turkey

**Keywords:** Dental caries, Multiple births, Oral habits, Singletons, Twins

## Abstract

**Objective:**

The aims of this study were to investigate and compare the prevalence of dental caries and the frequency of oral habits, molar relationships and occlusal traits between children of multiple births, and singletons, and to determine the relative contributions of genetics and environmental factors to these parameters by using twin study design.

**Methods:**

The study group consisted of 345 multiple births (34 monozygotic and 122 dizygotic twin pairs, 11 sets of triplets) and 345 singletons between the ages of 2 and 17. The prevalence of dental caries, and the frequency of tooth brushing, the children’s oral habits, molar relationships, and occlusal traits were recorded.

**Results:**

The percentage of children who brushed their teeth more than twice daily was statistically significantly higher in multiple births than in singletons. Higher correlation coefficients were found in dental caries index, except for decayed, filled (df) (2–5 age group) and filled (f) (6–11 age group), in the monozygotic twin pairs compared to those in the dizygotic twin pairs. In children between the ages of 6 and 11 years, mouth breathing, bruxism, lip biting, and pencil biting were higher in singletons than in children of multiple births. There were statistically significant differences between children of multiple births and singletons, with increased overjet in the 2–5 year age group being observed.

**Conclusion:**

When analyzing these parameters, environmental factors must also be investigated. Due to the low incidence of twin births, longitudinal follow-up studies with more twin pairs are necessary to determine whether these results are generalizable.

## Introduction

Oral health problems are common public health concerns in childhood and throughout life. It is important to understand the genetic basis of susceptibility to dental problems to take preventive measures earlier [1]. Twin studies help clinicians determine the contributions of genetic, epigenetic, and environmental factors to variations in dental traits [1, 2].

Dental caries occurrence and progression are influenced by several factors such as poor oral hygiene, inadequate salivary flow and composition, dietary habits, and tooth anatomy [3]. When analyzing the caries risk and incidence in twins, genetic contributions can be estimated by comparing similarity between monozygotic twins with that between dizygotic twins [4].

Dental caries affecting primary dentition lead to premature loss of primary teeth. Premature loss of primary teeth causes migration of adjacent teeth, which results in malocclusion [5]. Malocclusion is one of the most common diseases associated with dental caries, and it is predominantly the result of oral habits, meaning few orthodontists considered it to be hereditary [6].

The deleterious oral habits that affect oral and dental health have genetic or non-genetic origins. Certain habits, such as tongue thrust, thumb sucking, and bruxism, have been indicated in twin studies to be genetic traits [7, 8].

Although the assumption in twin studies is that their results may be reflected in the singleton population, the validity of this assumption has not been yet proven as there were studies reporting to the contrary due to differences of pre- and postnatal environments [9, 10]. Hence, this study was performed to fill the research gap by considering data collected in Turkey from singleton children and those of multiple births, all spanning a range of ages.

The Western Mediterranean Region of Turkey (covering the provinces of Antalya, Burdur, and Isparta), which constitutes the scope of this study, has a high population growth rate; it constitutes 4% of Turkey’s population. Antalya can be characterized as playing an important role in the development of Turkey’s health tourism in the region, largely consisting of people of the same ethnic origin, and leading other regions of Turkey in terms of socioeconomic development [11]. The children in this study benefited from public health services through general health insurance.

To our knowledge, this is the first comprehensive study of dental caries, oral habits, molar relationships, and occlusal traits focusing on different age groups of children of multiple births in Turkey.

In that context, the aims of this study were (1) to investigate and compare the prevalence of dental caries and the frequency of oral habits, molar relationships, and occlusal traits between children of multiple births and singletons, and (2) to also compare these factors between intrapair and interpair twins to gain an understanding of the relative contributions of genetics and environmental factors.

The null hypothesis (H0) for this research was that genetic influence on the parameters investigated in the study is zero. The alternative null hypothesis was that these parameters represent complex traits influenced by both genetics and environment.

## Materials and methods

### Ethical aspects

Ethics approval for the research was obtained from Suleyman Demirel University Faculty of Medicine Clinical Research Ethics Committee (2018/232) and registered with ClinicalTrials.gov (identification number: NCT04697017-06/01/2021). Informed consent to participate in the study was obtained from the participants’ parents. The study was reported according to the Strengthening The Reporting of Observational Studies in Epidemiology (STROBE) guidelines.

### Study samples

This study comprised 36 monozygotic and 125 dizygotic twin pairs, 11 sets of triplets, and 355 singletons between the ages of 2 and 17, all of whom attended the Department of Pediatric Dentistry at Suleyman Demirel University between January 2018 and September 2020. Children who had developmental anomalies such as cleft lip and palate, systemic diseases, premature tooth loss, or syndromes that could influence oral health, children who had already undergone or were undergoing orthodontic treatment, and children whose families did not give consent were excluded from the study.

Afterward, the study group consisted of multiple births (34 monozygotic and 122 dizygotic twin pairs, 11 set of triplets), and there were 345 singletons as a control group. Since there were parents in the triplet group who did not know the twin status of their children, 11 set of triplets only included in the multiple births group. When forming the control group, an effort was made to match multiple birth patients to singletons with similar parental education and socioeconomic status. Since the number of twin pairs was below certain limits, all twins who lived in the region and attended clinics for check-ups and/or dental treatments were evaluated according to the study’s inclusion criteria. For this reason, it was not possible to match monozygotic to dizygotic twins with similar parental education and socioeconomic status. To minimize the age effect, the participants were classified into three groups based on the dentition period (primary; 2 to 5 years of age, mixed; 6 to 11 years of age, permanent; 12 to 17 years of age). Sociodemographic details including age, gender, date of birth, and parental education were recorded.

### Clinical examination

The clinical examinations were performed by the same experienced pediatric dentist (E.O.). Dental caries status was recorded using the decayed, missing, and filled tooth (dmft) index for primary dentition and (DMFT) index for permanent dentition [12]. When evaluating patients over six years old, the missing tooth component was not included in the index, considering that teeth that were not located in the mouth might be physiologically exfoliated when aged over six.

Tooth brushing frequency was recorded as ‘’never’’, ‘’once a week or less’’, ‘’once a day’’, and ‘’ twice or more per day’’. The parents were asked about their children’s past and present oral habits (tongue thrust, bruxism, digit sucking, lip biting, cheek biting, nail biting, pencil biting, and mouth breathing) via a questionnaire. The patients were examined clinically for oral findings that could be attributable to those oral habits. In the diagnosis of bruxism (a movement disorder characterized by grinding and clenching of teeth), factors were considered that included anamnesis, dental clinical findings such as tooth wear, pain in the temporomandibular joint and mastication muscles, and headache. The breathing pattern (based on observation of the resting position) and clinical history (such as snoring at night, sleeping with open mouth, respiratory conditions) were evaluated to make a diagnosis of mouth breathing. For lip biting habit, the patients were examined for signs such as reddened/chapped lips and constant wetting of lips. Cheek biting habit was assessed clinically according to presence of hypertrophy of the buccal mucosa with a diffuse white, flaky appearance. For digit sucking habit, wrinkled, irritative eczema, callosities in all fingers were examined. The nail biting habit was evaluated by observation of fingernails recognizing the shape and contour of nails, the presence of damage to the cuticle and lesions in nail beds. To evaluate tongue thrusting habit, tongue activity during swallowing and tongue posture at rest by setting patient upright were examined.

The primary molar relationships were evaluated based on the flush terminal plane, mesial step, and distal step. The permanent molar relationships were recorded as Class I, Class II (Division 1 and 2), and Class III [13]. Occlusal traits such as increased overjet (≥ 3 mm distance of the most protruded maxillary incisor to the corresponding mandibular incisor), deep bite [excessive vertical overlapping (more than two thirds) of the mandibular incisors by the maxillary insicors in centric occlusion], open bite (a lack of vertical contact between the upper and lower teeth in the anterior region), anterior crossbite (lingual positioning of at least one maxillary incisor/canine in relation to the mandibular incisors/canine) and posterior crossbite (one or more of the maxillary molars occluded lingually to the mandibular molars) were recorded as present (1) or absent (0) with the help of a dental mirror.

To estimate the reliability of the measurement procedures, all variables from 10 randomly selected individuals were evaluated twice with a 1-week time interval. The clinical evaluations were performed by the same pediatric dentist (E.O.) (intraexaminer κ = 0.86) according to World Health Organization (WHO) instructions [11].

### Statistical analysis

The statistical analyses were performed using IBM SPSS V23 (Chicago, IL, USA). Conformity to normal distribution was evaluated using the Shapiro–Wilk and Kolmogorov–Smirnov tests, while Chi-Square and Fisher’s exact tests were used to compare categorical variables according to groups. In the comparison of quantitative variables according to twin groups, the Mann–Whitney U test was used for data that were not normally distributed. The results of the analyses were presented as median (minimum-maximum) for quantitative data and as frequency (percentage) for categorical data.

Spearman’s rho correlation coefficient (r) was used to examine relationships between non-normally distributed quantitative variables. The strength of the correlation was determined as; r < 0.30 ‘’weak’’, r = 0.30–0.59 was ‘’moderate’’, and r ≥ 0.60 was ‘’strong’’ [14]. In the classic twin method, Falconer’s formula [h^2^ = 2(rMZ- rDZ)] was used to estimate heritability (h^2^) for quantitative variables to understand the relative contribution of genetics to phenotypic variation with the value ranging from 0 (no heritability) to 1 [15]. The relative contribution of shared environmental effects (c^2^) was estimated according to c^2^ = 2rDZ-rMZ. The h^2^ value < 0.2 was considered as low heritability, >0.8 high heritability [16]. To explore the contribution of genetics vs. environment for categorical (qualitative) variables within twin pairs; pairwise concordance and tetrachoric correlation were used. Pairwise concordance and tetrachoric correlation analyses with 95% confidence intervals (Cls) for monozygotic and dizygotic twins were carried out using STATA software version 14.2 (StataCorp, College Station, TX, USA). The difference between the two correlation coefficients was computed by using STATISTICA software version 10.0 (StatSoft Inc, USA). The levels of significance were set at 5% (p < 0.05). If the p value was under 0.05 (p ≤ 0.05), results were considered statistically significant.

## Results

In this study, 156 twin pairs, 11 sets of triplets, and 345 singletons (control group) were included. 34 twin pairs were classified as monozygotic and 122 as dizygotic. The demographic characteristics of the study group were shown in Table 1.

**Table 1:** The demographic characteristics of the study group

Variables	MZ (n = 68) n (%)	DZ (n = 244) n (%)	Triplets (n = 33) n (%)	Singletons (n = 345) n (%)	M.births (n = 345) n (%)
**Range of age, years**	3.4–17.8	2.2–16.4	5.2–11.1	2.5-17.11	2.2–17.8
**Age in years, Mean (SD)**	8.10(3.30)	7.26(2.74)	7.53(2.10)	7.46(2.80)	7.45(2.82)
**Age groups**					
2–5	14(20.6)	96(39.3)	12(36.4)	122(35.4)	122(35.4)
6–11	44(64.7)	128(52.5)	21(63.6)	193(55.9)	193(55.9)
12–17	10(14.7)	20(8.2)	-	30(8.7)	30(8.7)
**Gender**					
Female	38(55.9)	126(51.6)	14(42.4)	178(51.6)	178(51.6)
Male	30(44.1)	118(48.4)	19(57.6)	167(48.4)	167(48.4)
**Mother education**					
Primary	28(41.2)	48(19.7)	9(27.3)	85(24.6)	85(24.6)
Secondary	6(8.8)	38(15.6)	-	44(12.8)	44(12.8)
High school	18(26.5)	86(35.2)	12(36.4)	116(33.6)	116(33.6)
University	16(23.5)	72(29.5)	12(36.4)	100(29.0)	100(29.0)
**Father education**					
Primary	20(29.4)	33(13.5)	9(27.3)	62(18.0)	62(18.0)
Secondary	12(17.7)	36(14.8)	-	48(13.9)	48(13.9)
High school	22(32.3)	85(34.8)	6(18.2)	113(32.7)	113(32.7)
University	14(20.6)	90(36.9)	18(54.5)	122(35.4)	122(35.4)

Abbreviations: MZ Monozygotic, DZ Dizygotic, SD Standard Deviation, M. births Multiple births

### Oral health status

The percentage of children who brushed their teeth more than twice daily was statistically significantly higher in multiple births (7.5%) than in singletons (1.2%) (p < 0.001). However, no statistically significant intrapair (p = 0.987 for monozygotic twin pairs, p = 0.755 for dizygotic twin pairs) or interpair (p = 0.430) relationships in tooth brushing frequency were found in monozygotic and dizygotic twins.

According to the dmft index, the highest median d values were observed in the 2–5-year-old group. There were statistically significant relationships in terms of median d and df values between children of multiple births and singletons in the 2–5 age group (p < 0.05). However, no differences were observed in the 6–11 and 12–17 age groups (Table 2).

**Table 2:** The median d, m, f, df, dmf values of the children in age groups

Age groups		2–5				6–11				12–17			
Parameters		MZ	DZ	M. births	Singletons	MZ	DZ	M.births	Singletons	MZ	DZ	M.births	Singletons
**d**	**Med (min-max)**	5 (0–11)	5 (0–17)	5 (0–17)	6.5 (0–20)	3 (0–16)	2 (0–14)	2 (0–9)	2 (0–20)	--	0 (0–1)	0 (0–1)	0 (0–3)
	**p-value**	0.914		*0.007		0.134		0.674		0.309		0.060	
**m**	**Med (min-max)**	0 (0–7)	0 (0–8)	0 (0–8)	0 (0–9)								
	**p-value**	0.600		0.310									
**f**	**Med (min-max)**	0 (0–8)	0 (0–7)	0 (0–8)	0 (0–9)	1 (0–7)	0 (0–7)	0 (0–7)	0 (0–10)	--	0 (0–1)	0 (0–1)	0 (0–3)
	**p-value**	0.448		0.567		0.299		0.615		0.309		0.584	
**df**	**Med (min-max)**	6 (4–11)	6 (0–17)	6 (0–17)	7 (0–20)	5 (0–16)	4 (0–14)	4 (0–16)	4 (0–20)	--	0 (0–2)	0 (0–2)	0 (0–5)
	**p-value**	0.408		*0.008		0.094		0.906		0.205		0.145	
**dmf**	**Med (min- max)**	7 (4–16)	6 (0–19)	6 (0–19)	8 (0–20)								
	**p-value**	0.279		0.054									
**D**	**Med (min-max)**					0 (0–4)	0 (0–10)	0 (0–10)	0 (0–10)	0 (0–6)	1 (0–9)	0 (0–9)	1 (0–8)
	**p-value**					0.968		0.998		0.172		0.164	
**M**	**Med (min-max)**									0 (0–3)	--	0 (0–3)	0 (0–1)
	**p-value**									0.157		0.981	
**F**	**Med (min-max)**					0 (0–3)	0 (0–3)	0 (0–4)	0 (0–6)	1 (0–11)	1 (0–6)	1(0–11)	0 (0–2)
	**p-value**					0.050		0.554		0.870		0.536	
**DMF**	**Med (min-max)**					0 (0–4)	0 (0–10)	0 (0–10)	0 (0–10)	1 (0–20)	3 (0–9)	2 (0–20)	2 (0–14)
	**p-value**					0.512		0.854		0.179		0.758	

Abbreviations: MZ Monozygotic, DZ Dizygotic, Med (Median), (min-max) (minimum–maximum), M. births Multiple births, d decay, m missing, f fiiled, df decay filled, dmf decay, missing, filled, Mann-Whitney U test ,* p < 0.05 values were statistically significant

Higher correlation coefficients (r-values) were found in dental caries index, except for df (2–5 age group) and f (6–11 age group), in the monozygotic twin pairs compared to those in the dizygotic twin pairs. Moderate heritability estimates (0.2 < h^2^ < 0.8) were recorded for all variables in 2–5 and 6–11 age groups, except for df (2–5 age group) and f, D (6–11 age group), with low heritability (h^2^ < 0.2). The highest heritability estimate (h^2^ = 1.460) was observed for F in the 12–17 age group (Table 3).

**Table 3:** Correlation coefficients (r), Genetic Heritability (h^2^), Cultural Inheritance (c^2^) and the differences of two correlation coefficients between twin members for parameters

Age groups	Parameters	rDZ (95% Cl)	rMZ (95% Cl)	h^2^ (95% Cl)	c^2^ (95% Cl)	rDZ/rMZ p value
**2–5**	**d**	0.612 (0.38, 0.77)	0.845 (0.10, 0.98)	0.466 (-0.56, 0.51)	0.379 (0.16, 0.43)	0.102
	**m**	0.601 (0.36, 0.77)	0.764 (-0.11, 0.97)	0.326 (-0.94, 0.40)	0.438 (0.23, 0.48)	0.331
	**f**	0.127 (-0.16, 0.40)	--	--	--	
	**df**	0.597 (0.36, 0.76)	0.514 (-0.44, 0.92)	-0.166 (-1.60, 0,32)	0.680 (0.16, 0.73)	0.707
	**dmf**	0.618 (0.38, 0.78)	0.745 (-0.14, 0.97)	0.254 (-1.04, 0.38)	0.491 (0.39, 0.52)	0.454
**6–11**	**d**	0.380 (0.14, 0.58)	0.719 (0.38, 0.89)	0.678 (0.48, 0.71)	0.041 (-0.1, 0.27)	*0.006
	**f**	0.638 (0.45, 0.77)	0.581 (0.18, 0.82)	-0.114 (-0.54, 0.10)	0.695 (0.12, 0.72)	0.615
	**df**	0.404 (0.17, 0.60)	0.703 (0.36, 0.88)	0.598 (0.38, 0.61)	0.105 (-0.02, 0.32)	*0.015
	**D**	0.507 (0.29, 0.68)	0.582 (0.18, 0.82)	0.150 (-0.22, 0.28)	0.432 (0.4, 0.54)	0.526
	**F**	0.306 (0.06, 0.52)	0.593 (0.19, 0.82)	0.574 (0.26, 0.60)	0.019 (-0.07, 0.22)	*0.044
	**DMF**	0.519 (0.30, 0.69)	0.664 (0.30, 0.86)	0.290 (0, 0.34)	0.374 (0.3, 0.52)	0.213
**12–17**	**d**	-0.111 (-0.69, 0.56)				
	**f**	-0.111 (-0.69, 0.56)				
	**df**	-0.167 (-0.72, 0.52)				
	**D**	0.525 (-0.20, 0.88)	--	--	--	
	**M**	--	--	--	--	
	**F**	0.031 (-0.61, 0.65)	0.761 (-0.52, 0.99)	1.460 (0.18, 1.48)	-0.699 (-0.73, 0.31)	*0.041
	**DMF**	0.678 (0.00, 0.93)	0.825 (-0.41, 0.99)	0.294 (-0.82, 0.32)	0.531 (0.41, 0.87)	0.447

Abbreviations: r Spearman’s rho correlation coefficient, MZ Monozygotic, DZ Dizygotic, Cl Confidence Interval, *p < 0.05 values were statistically significant

Genetic component: h^2^ = 2(rMZ - rDZ), Environmental component: c^2^ = 2rDZ–rMZ

### Oral habits

In the 2–5 age group, 13.1% and 25.4% of multiple birth and singleton children had bruxism, 0.8% and 10.7% had a lip-biting habit, and 5.7% and 0% had a digit-sucking habit, respectively, and statistically significant relationships were observed (p = 0.015, p = 0.001, p = 0.014, respectively). In children between the ages of 6 and 11 years, mouth breathing, bruxism, lip biting, and pencil biting were higher in singletons than in children of multiple births (p < 0.05). In children between the ages of 12 and 17 years, the rate of lip biting was higher in singletons compared to children of multiple births (p = 0.020). The rates of nail biting and bruxism were higher in dizygotic twins than in monozygotic twins in the 6–11 age group (p < 0.05). Higher pairwise concordance (r = 0.869, p < 0.001) and tetrachoric correlation (r = 0.999, p = 0.001) in terms of mouth breathing were observed among monozygotic twin pairs compared to dizygotic twin pairs between the ages of 6 and 11. This result suggested strong genetic effect for mouth breathing (Table 4).

**Table 4:** Comparison of the frequency of oral habits in the study group and correlations of oral habits between twin members

Age groups	Oral Habits	MZ n (%)	DZ n (%)	M.births n (%)	Singletons n (%)	Pairwise Concordance (95% Cl)				Tetrachoric correlation (95% Cl)					
						MZ pairs		DZ pairs		MZ pairs				DZ pairs	
						r	p value	r	p value	r			p value	r	p value
**2–5**	**Mouth breathing**	1(7.1)	16(16.7)	18(14.8)	27(22.1)	-		0.271	0.062	-				0.495	0.095
	**p value**	0.692^ F^		0.137				(0.07, 0.47)						(0.32, 0.67)	
	**Nail biting**	3(21.4)	13(13.5)	21(17.2)	20(16.4)	0.645	0.117	0.379	*0.008	0.999			0.286	0.644	*0.033
	**p value**	0.426^ F^		0.864		(0.16, 1.12)		(0.19, 0.57)		(0.90–1.08)				(0.49, 0.80)	
	**Bruxism**	1(7.1)	13(13.5)	16(13.1)	31(25.4)	-		0.030	0.837	-				0.074	1.000
	**p value**	0.691^ F^		*0.015				(-0.18, 0.24)						(0.6, 0.88)	
	**Lip biting**	1(7.1)	0(0)	1(0.8)	13(10.7)	-		-		-				-	
	**p value**	--		*0.001											
	**Cheek biting**	0(0)	1(1)	1(0.8)	1(0.8)	-		-		-				-	
	**p value**	--		1.000^ F^											
	**Pencil biting**	0(0)	0(0)	1(0.8)	2(1.6)	-		-		-				-	
	**p value**	--		1.000^ F^											
	**Tongue thrust**	0(0)	0(0)	0(0)	1(0.8)	-		-		-				-	
	**p value**	--		--											
	**Digit sucking**	1(7.1)	6(6.3)	7(5.7)	0(0)	-		0.999	*<0.001	-				0.999	*<0.001
	**p value**	1.000^ F^		*0.014^ F^				(0.96, 1.02)						(0.96, 1.02)	
**6–11**	**Mouth breathing**	9(20.5)	23(18)	32(16.6)	48(24.9)	0.869	*<0.001	0.125	0.323	0.999			*0.001	0.252	0.377
	**p value**	0.715		*0.045		(0.72, 1.02)		(-0.05, 0.30)		(0.95, 1.03)				(0.08, 0.42)	
	**Nail biting**	4(9.1)	29(22.7)	35(18.1)	37(19.2)	-0.087	0.701	0.064	0.615	-0.999		1.000		0.120	0.723
	**p value**	*0.049		0.794		(-0.40, 0.22)		(-0.11, 0.24)		(-1.03,-0.95)				(-0.05, 0.29)	
	**Bruxism**	3(6.8)	28(21.9)	31(16.1)	51(26.4)	-		0.114	0.371	-				0.216	0.448
	**p value**	*0.025		*0.013				(-0.06, 0.29)						(0.05, 0.39)	
	**Lip biting**	3(6.8)	8(6.3)	12(6.2)	36(18.7)	0.690	*<0.001	-0.058	0.650	0.999	0.091			0.298	1.000
	**p value**	1.000^ F^		*<0.001		(0.46, 0.92)		(-0.23, 0.12)		(0.95, 1.03)				(0.13, 0.47)	
	**Cheek biting**	0(0)	1(0.8)	1(0.5)	5(2.6)	-		-		-				-	
	**p value**	--		0.215^ F^											
	**Pencil biting**	6(13.6)	8(6.3)	16(8.3)	31(16.1)	0.261	0.241	0.211	0.094	0.506	0.338			0.496	0.220
	**p value**	0.196^ F^		*0.020		(-0.04, 0.56)		(0.04, 0.38)		(0.24, 0.78)				(0.34, 0.65)	
	**Tongue thrust**	1(2.3)	1(0.8)	2(1)	7(3.6)	-		-		-				-	
	**p value**	0.447^ F^		0.092											
	**Digit sucking**	1(2.3)	2(1.6)	3(1.6)	1(0.5)	-		-		-				-	
	**p value**	1.000^ F^		0.623^ F^											
**12–17**	**Mouth breathing**	1(10)	4(20)	5(16.7)	1(3.3)	-		0.509	0.133	-				0.299	0.647
	**p value**	0.640^ F^		0.195^ F^				(0.08, 0.94)						(-0.17, 0.77)	
	**Nail biting**	3(30)	1(5)	4(13.3)	8(26.7)	0.612	0.272	-		0.999	0.400			-	
	**p value**	0.095^ F^		0.197		(-0.01, 1,23)				(0.92, 1.06)					
	**Bruxism**	1(10)	3(15)	4(13.3)	5(16.7)	-		-		-				-	
	**p value**	1.000^ F^		1.000^ F^											
	**Lip biting**	1(10)	1(5)	2(6.7)	9(30)	-		-		-				-	
	**p value**	1.000^ F^		*0.020											
	**Cheek biting**	0(0)	0(0)	0(0)	0(0)	-		-		-				-	
	**p value**	--		--											
	**Pencil biting**	0(0)	3(15)	3(10)	3(10)	-		0.667	*0.035	-				0.481	0.200
	**p value**	0.532^ F^		1.000^ F^				(0.30, 1.04)						(0.05, 0.92)	
	**Tongue thrust**	0(0)	0(0)	0(0)	1(3.3)	-		-		-				-	
	**p value**	--		--											
	**Digit sucking**	0(0)	0(0)	0(0)	0(0)	-		-		-				-	
	**p value**	--		--											

Abbreviations: M.births Multiple births, MZ Monozygotic, DZ Dizygotic, Chi-square test, F: Fisher’s Exact test, *p < 0.05 values were statistically significant

### Occlusal measures

Statistically significant differences in molar classification (for the 2–5 age group in terms of the flush terminal plane, mesial step and distal step; for the 6–11 age group in terms of Class I, Class II Div 1, Class II Div 2, and Class III) were observed between monozygotic and dizygotic twins and between children of multiple births and singletons in the 2–5 and 6–11 age groups (p < 0.05). The pairwise concordances and tetrachoric correlations for the flush terminal plane and distal step were statistically significant in dizygotic twin pairs between the ages of 2 and 5 (p < 0.001). This result showed the effects of environmental factors on flush terminal plane and distal step parameters. Higher pairwise concordances and tetrachoric correlations for Class I and II molar relationships were determined within monozygotic twin pairs compared to dizygotic twin pairs in the 6–11 age group (p < 0.001). The higher correlations in the MZ compared with DZ twins indicated the moderate influence of genetic factor. However, the statistically significant relationships observed in monozygotic twin pairs were not valid for the 2–5 age group (Table 5).

**Table 5:** Comparison of molar relationships in the study group and correlations of molar relationships between twin members

Age groups	Molar Classification	MZ n (%)	DZ n (%)	p value	M.Births n (%)	Singletons n (%)	p value	Pairwise Concordance (95% Cl)				Tetrachoric correlation (95% Cl)			
								MZ pairs		DZ pairs		MZ pairs		DZ pairs	
								r	p value	r	p value	r	p value	r	p value
**2–5**	**Flush terminal**	6(42.9)	56(58.3)	*<0.001	74(60.7)	84(68.9)	*0.044	0.558	0.203	0.829	*<0.001	0.999	0.429	0.964	*<0.001
								(0.04, 1.07)		(0.71, 0.94)		(0.96, 1.02)		(0.91, 1.02)	
	**Mesial step**	5(35.7)	4(4.2)		9(7.4)	15(12.3)		0.730	0.062	-0.043	0.769	0.999	0.143	0.538	1.000
								(0.31, 1.15)		(-0.25, 0.16)		(0.96, 1.02)		(0.37, 0.71)	
	**Distal step**	3(21.4)	36(37.5)		39(32)	23(18.9)		0.645	0.117	0.644	*<0.001	0.999	0.286	0.852	*<0.001
								(0.16, 1.13)		(0.49, 0.80)		(0.96, 1.02)		(0.74, 0.96)	
**6–11**	**Class I**	17(38.6)	37(28.9)	*<0.001	55(28.5)	80(41.5)	*0.005	0.908	*<0.001	0.506	*<0.001	0.999	*<0.001	0.732	*<0.001
								(0.78, 1.04)		(0,36, 0.66)		(0.97, 1.02)		(0.61, 0.85)	
	**Class II**	15(34.1)	66(51.6)		94(48.7)	66(34.2)		0.904	*<0.001	0.505	*<0.001	0.999	*<0.001	0.717	*<0.001
								(0.78, 1.04)		(0.35, 0.66)		(0.97, 1.02)		(0.60, 0.84)	
	**Class II Div 1**	0(0)	18(14.1)		21(10.9)	21(10.9)		-		0.371	*0.002	-		0.642	*0.013
										(0.21, 0.53)				(0.51, 0.78)	
	**Class II Div 2**	4(9.1)	1(0.8)		5(2.6)	14(7.3)		0.999	*<0.001	-		-		-	
								(0.95, 1.03)							
	**Class III**	8(18.2)	6(4.7)		18(9.3)	12(6.2)		0.999	*<0.001	0.325	*0.009	-		0.684	0.122
								(0.95, 1.03)		(0.16, 0.49)				(0.56, 0.81)	
**12–17**	**Class I**	2(20)	4(20)	0.874	6(20)	10(33.3)	0.228	0.999	*<0.001	0.509	0.133	-		0.299	0.300
								(0.88, 1.10)		(0.08, 0.94)				(-0.17, 0.77)	
	**Class II**	6(60)	10(50)		16(53.3)	15(50)		0.408	0.495	0.200	0.580	-		0.309	1.000
								(-0.34, 1.15)		(-0.29, 0.69)				(-0.16, 0.78)	
	**Class II Div 1**	2(20)	5(25)		7(23.3)	3(10)		-		0.218	0.545	-		0.375	1.000
										(-0.27, 0.70)				(-0.08, 0.83)	
	**Class II Div 2**	0(0)	1(5)		1(3.3)	0(0)		-		-		-		-	
	**Class III**	0(0)	0(0)		0(0)	2(6.7)		-		-		-		-	

Abbreviations: M.births Multiple births, Div Division, MZ Monozygotic, DZ Dizygotic, Chi-square test, *p < 0.05 values were statistically significant

When the occlusal traits were evaluated, statistically significant relationships in anterior crossbite were found between monozygotic and dizygotic twins in the 2–5 and 6–11 age groups (p < 0.05). There were also statistically significant differences between children of multiple births and singletons, with increased overjet in the 2–5 year age group and posterior crossbite in the 6–11 age group both observed (p < 0.05). Higher pairwise concordances and tetrachoric correlations for anterior crossbite were observed within monozygotic twin pairs in the 2–5 and 6–11 age groups. This indicated genetic influence. The pairwise concordance (r = 0.700, p < 0.001) and tetrachoric correlation (r = 0.913, p < 0.001) for increased overjet were statistically significant in dizygotic twin pairs between the ages of 2 and 5. Higher pairwise concordance (r = 0.999, p < 0.001) and tetrachoric correlation (r = 0.999, p = 0.045) in terms of anterior open bite were determined within monozygotic twin pairs compared to dizygotic twin pairs in the 6–11 year age group (p < 0.05) (Table 6). The monozygotic correlations were higher than dizygotic correlations in this feature, suggesting the influence of genetic factor.

**Table 6:** Comparison of occlusal traits in the study group and correlations of occlusal traits between twin members

Age groups	Occlusal traits	MZ n (%)	DZ n (%)	M.births n (%)	Singletons n (%)	Pairwise Concordance (95% Cl)						Tetrachoric correlation (95% Cl)				
						MZ pairs			DZ pairs			MZ pairs			DZ pairs	
						r	p value		r	p value		r	p value		r	p value
**2–5**	**Anterior crossbite**	4(28.6)	1(1)	5(4.1)	5(4.1)	0.999	*<0.001		-			0.999	0.048		-	
	**p value**	*0.001^ F^		1.000		(0.90, 1.08)						(0.90, 1.08)				
	**Posterior crossbite**	1(7.1)	5(5.2)	6(4.9)	1(0.8)	-			-0.044	0.767		-0.044	0.767		0.576	1.000
	**p value**	0.567^ F^		0.120^ F^					(-0.25, 0.16)			(-0.66, 0.58)			(0.41, 0.74)	
	**Anterior openbite**	0(0)	3(3.1)	3(2.5)	1(0.8)	-			0.377	*0.008		0.377	*0.008		0.721	0.122
	**p value**	1.000^ F^		0.622^ F^					(0.19, 0.57)			(-0.20, 0.95)			(0.58, 0.86)	
	**Deep bite**	0(0)	1(0)	0(0)	1(0.8)	-			-			-			-	
	**p value**	--		--												
	**Increased overjet**	1(7.1)	13(13.5)	15(12.3)	6(4.9)	-0.258	0.576		0.700	*<0.001		-			0.913	*<0.001
	**p value**	0.691^ F^		*0.040		(-0.86, 0.34)			(0.55, 0.85)						(0.83, 0.99)	
**6–11**	**Anterior crossbite**	5(11.4)	2(1.6)	10(5.2)	19(9.8)	0.796	*<0.001		-			0.999		*0.013	-	
	**p value**	*0.013^ F^		0.082		(0.61, 0.99)						(0.97, 1.01)				
	**Posterior crossbite**	4(9.1)	4(3.1)	12(6.2)	1(0.5)	0.549		*0.008	-			0.999		0.136	-	
	**p value**	0.206^ F^		*0.002		(0.29, 0.81)						(0.97, 1.01)				
	**Anterior openbite**	0(0)	3(2.3)	3(1.6)	3(1.6)	0.999		*<0.001	-0.028	0.826		0.999		*0.045	0.669	1.000
	**p value**	0.571^ F^		1.000^ F^		(0.95, 1.03)			(-0.20, 0.15)			(0.97, 1.01)			(0.54, 0.80)	
	**Deep bite**	6(13.6)	8(6.2)	14(7.2)	15(7.2)	0.999		*<0.001	0.488	*<0.001		-			0.813	*0.005
	**p value**	0.196^ F^		0.847		(0.95, 1.03)			(0.34, 0.64)						(0.71, 0.92)	
	**Increased overjet**	3(6.8)	18(14.1)	24(12.4)	27(14.0)	0.796		*<0.001	0.417	*<0.001		-			0.640	*0.002
	**p value**	0.205		0.652		(0.61, 0.99)			(0,26, 0.58)						(0.51, 0.77)	
**12–17**	**Anterior crossbite**	0(0)	0(0)	0(0)	5(16.7)				-						-	
	**p value**	--		0.052^ F^												
	**Posterior crossbite**	0(0)	0(0)	0(0)	1(3,3)				-						-	
	**p value**	--		--												
	**Anterior openbite**	0(0)	0(0)	0(0)	0(0)				-						-	
	**p value**	--		--												
	**Deep bite**	0(0)	1(2)	1(3.3)	1(3.3)	-			-			-			-	
	**p value**	--		--												
	**Increased overjet**	2(20)	5(25)	7(23.3)	7(23.3)	0.612		0.272	0.102		0.779	-			0.181	1.000
	**p value**	1.000^ F^		1.000^ F^		(-0.03, 1.26)			(-0.39, 0.60)						(-0.31, 0.67)	

Abbreviations: M.births Multiple births, MZ Monozygotic, DZ Dizygotic, *p < 0.05 values were statistically significant, Chi-square test, F: Fisher’s Exact test

It was not possible to estimate the pairwise concordances and tetrachoric correlations for monozygotic and dizygotic twins for some variables of oral habits and occlusal measures due to lack of numbers (Tables 4, 5 and 6).

## Discussion

### Oral health status

Twins who are raised together are likely to have similar oral hygiene practices and share the same habits during the first years of their lives [17]. In this study, the intrapair similarity of the tooth brushing frequency in both monozygotic and dizygotic twins showed that a common or shared environment (parenting style, social class) may have an impact on this practice. In addition, the finding that brushing the teeth twice or more per day was more likely in children of multiple births than in singletons may be attributed to how children of similar ages imitate each other by modeling.

Twin studies have also estimated that primary dentition caries have higher heritability than permanent dentition caries [18]. The highest incidence of dental caries occurs at an early age in primary dentition, that is, between the ages of 1.5 and 4 years [2]. Newly erupted teeth may be prone to caries development and progression because all of the deciduous tooth crown surfaces are already mineralized during the prenatal and early postnatal periods; thus, there is limited time for environmental factors to take effect [2, 19]. This was supported by our study, in which the highest median d values were observed in the 2–5-year-old group (Table 2).

Furthermore, higher r values for index associated with dental caries [except for df (2–5 age group) and f (6–11 age group)] in the monozygotic compared to the dizygotic twin pairs reflected a genetic influence. The genetic effect was explained by the ability of immune complex genes to modulate the presence of enamel defects and colonization of cariogenic bacteria [20]. Beyond that, the mostly moderate and strong correlation coefficients for caries-related features within dizygotic twin pairs in the 2–5 and 6–11 age groups also suggested the effects of environmental factors, as did the moderate (h^2^ ranged from 0.254 to 0.678) and low (h^2^ ranged from − 0.166 to 0.150) heritability potential of dental caries index in 2–5 and 6–11 age groups. Environmental (including behavioural) factors such as the differences in the education and socioeconomic status of parents (such as the higher percentage of parents in this study with high school and university education in dizygotic twins than monozygotic twins), along with the dietary habits, access to dental care of twins could affect the progression and prevalence of dental caries. Although there are similiar environmental aspects due to the twins being raised together, the results of study demonstrated the importance of both genetic and environmental influences on dental caries.

The highest h^2^ for F in monozygotic twin pairs in the 12–17 age group showed that the parameter was subject to a potential genetic influence, going against the notion that a greater age is linked with lower genetic influence because environmental factors have had longer to take effect. The heritability estimate of less than 0 and greater than 1 may reflect errors due to the small sample size, therefore to estimate the exact relative contributions of genetics and environment is difficult. Furthermore, h^2^ does not estimate the impact of the common environment, meaning the true values may be at the upper limits of those recorded [21].

The results of this study also showed that singletons had an increased caries rate compared to children of multiple births in the 2–5-year age group, though it was determined that the children may have had low median m and f values since caries were in the initial stage and treatments for decayed teeth were minimal in this age group.

### Oral habits

In the study, higher correlations of mouth breathing among monozygotic twin pairs compared to dizygotic twin pairs between the ages of 6 and 11 indicated a genetic contribution to the undetermined etiology of mouth breathing, potentially involving changes in the pattern of dento-skeletal growth and child development [22]. In addition, the lower correlations for dizygotic twins compared to monozygotic twins might indicate sex-specific influences. Correlation analyses were not performed for some oral habits with low sample frequency, which meant we could not interpret the contributions of genetic and environmental factors to the presence of these oral habits.

The statistically significant differences in bruxism and lip-biting habits between children of multiple births and singletons in the 2–5 and 6–11 age groups may be attributed to the similarity in responses to environmental stimuli of children of the same age, as occurs in multiple births. Beyond that, the increase in the frequency of oral habits in both children of multiple births and singletons between the ages of 6 and 11, when children begin socialization and school, may have been related to the individual (gender, low self-esteem, insufficient social relations, chronic diseases), familial (divorce of parents, death, disease, family conflict affecting the child), and environmental (stressful and traumatic events at school, rejection of peers, economic difficulties, low class) risk factors [23]. The fact that children of multiple births share the same environmental risk factors with their siblings may cause certain oral habits to be less common than they are in singletons.

### Occlusal measures

The interaction of genetic and environmental factors can cause occlusal variation in the development of the orofacial region [24]. In this study, the flush terminal plane and distal step relationships were highly significant within dizygotic twin pairs, indicating strong environmental influences such as early loss of primary teeth, oral habits, trauma, and interproximal caries on the development of molar relationships. However, a high rate of intrapair concordance of primary molar relationships in a study conducted on 3–5-year-old monozygotic twins indicated that there was a large genetic contribution, in contrast to the results obtained in our study [25]. The differences in the obtained results may arise from differences in the studied populations (e.g., ethnic background), study designs (twins vs. family studies) and the statistical methods used. In addition, inferring potential genetic influences on twins raised together was difficult in this study because the monozygotic twin sample size was small.

In terms of molar relationships, heritability may play an important role in affecting the etiology of Class I and II molar relationships in twins aged 6–11 years, given the higher correlations in monozygotic twin pairs than in dizygotic twin pairs. The similarity of malocclusions in twin pairs may be due to their similar craniofacial forms, which are more genetically determined [26].

Among the occlusal traits, anterior crossbite is associated with a prognathic skeletal pattern, and functional ones can favor a Class III growth pattern [27]. Crossbite has been explained to be the most prominent hereditary criterion of occlusion [28]. Accordingly, in this study, higher correlations in monozygotic twin pairs aged 2–5 and 6–11 years suggest that genetic factors play an important role. This contrasts with the research by Potter et al. (1981), who found a stronger environmental contribution to the development of crossbites [29].

Overjet, meanwhile, has a relatively lower heritability, with a strong environmental contribution instead confirmed [21, 24, 30]. In this study, we observed higher tetrachoric correlations for increased overjet in dizygotic twin pairs aged 2–5 years, indicating potential environmental effects on this trait, such as through long-term mouth breathing, prolonged thumb sucking, hypertrophic tonsils, and nasal blockage.

Anterior open bite is a multifactorial condition caused by the interaction of environmental factors (habits, trauma or pathology to one or both condyles, tooth loss, nasal obstruction, hypertrophic tonsils, neuromuscular deficiencies) and genetic factors [6]. Statistically significant correlations for anterior open bite were found in this study in monozygotic twin pairs compared to dizygotic twin pairs between the ages of 6 and 11, showing that there is limited environmental influence, with a strong genetic control such as inherited vertical facial growth pattern, abnormal tongue posture and size. Based on the results of twin pairs, occlusal traits concerning the relationship between the maxilla and the mandible have the environmental and hereditary components varing by age groups. In addition, a better understanding of the influences of genetic and environmental factors on these occlusal traits could help dentists in treatment planning.

When children in the 2-5-year-age were evaluated, overjet was higher in children of multiple births than in singletons. Parents may use bottles and pacifiers more when they have twins or triplets than when they have a single child since it is more difficult for them to care for children; accordingly, an environmental factor can be proposed to have contributed to this occlusal feature.

This study has some limitations to note, chiefly its small sample size and cross-sectional study design. The small sample size (with low or no patients with certain characteristics) limited the precision of some estimates and caused many estimates to be missing. Furthermore, the increase in multiple births in recent years has led to a low number of postpubescent children. Accordingly, the number of children in each age group differed, which may have affected the results and prevented the findings from being extrapolated. Moreover, to understand the heritability of oral health, oral habits, and occlusal traits, it is extremely important to investigate family-related, psychological, and microbial factors. Possible differences in tis regard (such as parenteral education and socioeconomic status) between monozygotic and dizygotic twin groups we sampled may have influenced the findings of this study. In addition, our failure to include radiographs in dental examinations may have meant some carious lesions and restorations were undetected, particularly on the approximal surfaces. In a further limitation, the genetic differences according to sex were not analyzed, and inaccuracies in the precision of zygosity determination may have influenced the findings, with zygosity between twin pairs was not confirmed by Deoxyribonucleic Acid (DNA) testing or other methods in this study.

Nonetheless, this study provides novel results for a single geographic location and ethnic origin in a context where it is difficult to form twin samples and complex to conduct this type of research, as a consequence of which there are limited published studies from the country. Adding to the extant literature, this study provides insight into how genetics can contribute to dental caries, oral habits, and occlusal traits in twins.

## Conclusion

As a result, some components investigated in this study were found to have underlying environmental factors, some had possibly of genetic origins, and this situation varied according to age group. When analyzing these parameters, the contributions of genetic and environmental factors must be investigated.

Due to the relatively low prevalence of twin births, longitudinal follow-up studies with more twin pairs should be conducted to determine whether these results are generalizable more precisely.

## Data Availability

All data generated or analysed during this study are included in this published article.
